# The interplay of emotional intelligence and mental toughness in amateur soccer: the role of goal commitment

**DOI:** 10.3389/fpsyg.2025.1670502

**Published:** 2025-11-19

**Authors:** Kadir Şekerci, Murat Şahbudak, Mustafa Enes Işıkgöz

**Affiliations:** 1 Şırnak Anatolian High School, Şırnak, Türkiye; 2 Faculty of Sports Sciences, Batman University, Batman, Türkiye; 3 Department of Rectorate, Sakarya University, Sakarya, Türkiye

**Keywords:** amateur soccer players, emotional intelligence, goal commitment, mental toughness, mediation

## Abstract

**Background:**

Emotional intelligence and mental toughness are recognized as key psychological factors influencing athletic performance and well-being. However, the mechanisms underlying the relationship between these constructs, and the potential impact of demographic factors, remain underexplored, especially among amateur soccer players.

**Purpose:**

The purpose of this study is to examine the mediating role of goal commitment in the relationship between emotional intelligence and mental toughness in amateur soccer players, and to determine whether this role is moderated by demographic factors.

**Methods:**

The study included 262 licensed amateur soccer players in Şırnak province, southeastern Türkiye, using a cross-sectional design and a correlational survey model. Data were collected with the Emotional Intelligence Scale, the Goal Commitment Scale, and the Mental Toughness Inventory in Sports. Analyses were conducted using Jamovi software (version 2.4.8) and the Bootstrap 5,000 resampling method.

**Results:**

The results indicated moderate positive relationships among emotional intelligence, goal commitment, and mental toughness. Mediation analysis indicated that goal commitment partially mediated the association between emotional intelligence and mental toughness; approximately 24% of the total association was indirect via goal commitment. Moderator analysis indicated that the indirect association varied by gender, athlete’s license year, and educational status influenced this mediation, while marital status did not.

**Conclusion:**

These findings suggest that goal commitment is importantly associated with emotional intelligence and mental toughness among amateur soccer players. The results highlight the need to consider individual differences and demographic factors such as gender, athlete license year, and educational status for designing interventions aimed at supporting performance and mental toughness in this population.

## 1 Introduction

In sports psychology, it is increasingly recognized that psychological factors are as crucial to performance as physical ability. Amateur athletes, who have fewer resources and support systems than professional athletes, must balance personal motivation, team dynamics, and daily life stressors. Emotional intelligence (EI) is defined as the ability to recognize and manage one’s own emotions and those of others (Laborde et al., 2018). In amateur soccer, EI is significantly associated with performance, as it is linked to improved team dynamics, stress management, and conflict resolution. Goal commitment (GC) improves communication among team members, enhancing trust and collaboration. Teams with strong emotional bonds and shared goals demonstrate better performance due to mutual support (Choudhury and Das, 2024). Additionally, higher EI has been linked to greater use of coping strategies for stress management (Lopes, 2024). Research shows that athletes with high EI experience lower levels of competition anxiety, which correlates with better concentration and performance during competitions (Rubio and Ángel, 2023; Zoghlami et al., 2023). Despite these benefits, some researchers argue that physical skills and technical training remain the primary determinants of success, emphasizing the need for a balanced approach that integrates psychological and physical training.

Goal commitment (GC) is also considered a key factor for success. It reflects an athlete’s determination to achieve their goals and the sustained effort they invest in reaching them (Locke and Latham, 2013). Research suggests that GC is positively associated with improved training discipline and perseverance, especially in amateur athletes whose participation is driven by intrinsic motivation (Özcan and Göral, 2020; Şenel and Ulaş, 2022). Furthermore, the literature indicates that GC can serve as a significant mediator in the relationship between training discipline and performance outcomes and is linked to increased resilience in the face of challenges (Çekiç and Kurt, 2017; Orhan and Ünlü, 2022; Weinberg and Gould, 2024; Yılmaz and Bostancı, 2024). GC is also known to influence athletes’ emotional responses to success and failure, thereby shaping their overall motivation and performance levels (Dewar and Kavussanu, 2012). Evidence links effective goal-setting strategies with greater focus and commitment (Weinberg and Butt, 2014). However, excessive focus on goals can be linked to stress and anxiety. Therefore, a balance among GC, EI, and coping strategies is considered crucial for optimal athletic performance (Özcan and Göral, 2020).

Beyond EI and GC, mental toughness (MT) is central to managing challenges ranging from injuries to competitive pressure. It reflects an athlete’s capacity to tolerate adversity, recover from setbacks, and make sound decisions under pressure. This toughness is a fundamental element for sustained performance and overall mental well-being (Mahoney et al., 2014; Dacus et al., 2023). Research shows that MT is correlated with an improved ability to perform under pressure (Özsoy, 2023). Athletes with high MT typically cope better with stress and maintain their motivation, which is crucial for success in competition (Kumbar and Patil, 2024). They also tend to show greater cognitive flexibility in high-pressure situations, enabling them to make better decisions (Fan and Zhang, 2019).

The interplay among these constructs is central. EI is associated with greater focus on long-term performance goals and stronger GC, and, under stress, higher EI co-occurs with MT linked to more effective coping (Laborde et al., 2018; Montenegro-Bonilla et al., 2024). This suggests a potential sequential pattern in which EI is associated with higher GC, which may, in turn, be associated with greater MT. Recent research also suggests that these relationships may vary across demographic characteristics. For example, women often report higher scores than men on empathy and sensitivity to emotional cues (Kumar et al., 2025). Additionally, factors such as age, educational attainment, and years of experience have been reported to influence both EI and MT (Luo et al., 2018). Therefore, examining the potential moderating roles of demographic variables is important for both theory and practice.

However, there is limited empirical evidence on how these relationships function specifically in amateur soccer players (Kipp and Bolter, 2024). In particular, further research is needed to determine whether GC serves as a mediating factor in the relationship between EI and MT, and whether demographic variables (e.g., gender, years of experience, educational level) moderate this process (Rogaleva et al., 2024; Beniwal and Yadav, 2025). This study investigates the relationships among EI, GC, and MT in amateur soccer players. It also examines the potential mediating role of GC in these associations and the moderating roles of selected demographic factors in this mediation. The results are expected to inform the development of strategies aimed at supporting amateur athletes’ performance and psychological resilience. This study focuses on amateur soccer players in Türkiye and is expected to make significant contributions to the sport psychology literature from both theoretical and practical perspectives, considering the cultural context.

## 2 Conceptual and theoretical framework

### 2.1 Emotional intelligence and its role in sports psychology

Emotional intelligence (EI) is defined as a person’s ability to recognize, understand, manage, and effectively reflect both their own emotions and the emotions of others in their behavior (Salovey and Mayer, 1990). This concept, developed by Salovey and Mayer (1990), emphasizes the ability to accurately perceive, evaluate, and express emotions, and has been identified by Goleman (2005) as a fundamental competence in work, educational, and social life. EI is considered a multidimensional psychological skill that enhances a person’s success in coping with stress, maintaining motivation, and being effective in social relationships and leadership. Theoretically, EI can be explained by various models. The ability model by Mayer and Salovey (1997) addresses EI through four fundamental components: perceiving emotions, using emotions, understanding emotions, and managing emotions. This model highlights that EI is integrated with cognitive processes and enables individuals to analyze both their own and others’ emotional states and develop appropriate responses. Goleman (1998)'s hybrid model examines EI in five basic dimensions: self-awareness, self-management, motivation, empathy, and social skills. This model suggests that EI is not only cognitive but also related to personality traits and social skills. Bar-On (1997)'s model focuses on social–emotional skills such as stress management, adaptability, and interpersonal relationships.

In the sport psychology literature, emotional intelligence (EI) is increasingly recognized as a pivotal factor influencing athletic performance, particularly in team sports (Laborde et al., 2016, 2018; Montenegro-Bonilla et al., 2024). Group cohesion (GC) enhances athletes’ abilities to manage stress, communicate effectively, and demonstrate leadership, all of which are essential for success in competitive environments (Rubio et al., 2022). Athletes with high EI are better equipped to handle anxiety, maintain focus, and resolve conflicts, leading to improved performance outcomes (Kotsou et al., 2018; Kopp et al., 2021; Beniwal and Yadav, 2025). Research also shows that EI contributes to cognitive processes crucial for decision-making and performance under pressure, while supporting effective communication and fostering team cohesion (Kopp et al., 2021; Montenegro-Bonilla et al., 2024). Although the benefits of EI in sports are well documented, some researchers suggest that its impact may vary depending on individual differences and the specific context of the sport, highlighting the need for further exploration into how EI interacts with other psychological factors (Kaygusuz, 2024).

The importance of emotional intelligence (EI) is even more evident among amateur footballers. Compared to professional athletes, amateurs generally have less psychological support, fewer resources, and less structured training programs, so they must rely more on their EI to cope with stress, uncertainty, and team conflict (Vyas and Khanvilkar, 2024; Solmaz and Şimşek, 2025). Recent studies indicate that EI plays a crucial role in team cohesion, maintaining motivation, and improving the individual performance of amateur soccer players (Choudhury and Das, 2024; Lopes, 2024). In amateur leagues, athletes with high EI are more successful in maintaining training consistency, coping with stress during matches, and building healthy relationships with teammates. In summary, EI is a key correlate of psychological resilience, motivation, and sustained performance at both the individual and team levels in amateur soccer players. Therefore, developing EI in amateur soccer players is considered a key strategy for sporting success and psychological well-being.

### 2.2 Goal commitment and its impact on athletic performance

Goal commitment (GC) is defined as an athlete’s determination to achieve their objectives and the extent to which they consistently strive toward these goals (Locke and Latham, 2013). In sports psychology, GC is recognized as a key motivational correlate associated with sustained athletic performance (Weinberg and Butt, 2014; Gucciardi et al., 2021). Closely linked to Goal Setting Theory, GC is most effective when athletes set SMART goals– specific, measurable, achievable, relevant, and time-bound– which have been shown to enhance motivation, focus, and overall success (Doran, 1981; Locke and Latham, 1990; Weinberg and Gould, 2024). In the sporting context, goals are typically categorized into three dimensions: task-oriented (focused on personal development and skill acquisition), performance-oriented (aimed at outperforming others), and outcome-oriented (centered on winning or achieving a specific result) (Weinberg and Butt, 2014; Moran, 2016).

High GC plays a crucial psychological role in promoting regular training participation, long-term performance development, and resilience in the face of competition-related challenges (Weinberg et al., 2001; Weinberg and Butt, 2014; Gucciardi et al., 2017, 2021; Weinberg and Gould, 2024). This is particularly significant for amateur soccer players, who often lack external rewards and professional support systems; for them, GC is a primary driver of intrinsic motivation and personal satisfaction (Özcan and Göral, 2020; Solmaz and Şimşek, 2025). Research consistently demonstrates that athletes with strong GC show greater training discipline, enhanced MT, and faster recovery from setbacks (Çekiç and Kurt, 2017; Kopp et al., 2021). Moreover, these athletes tend to maintain stable performance and motivation even under stressful, high-pressure conditions (Gucciardi et al., 2021; Orhan and Ünlü, 2022; Yılmaz and Bostancı, 2024).

However, it is important to recognize that excessive goal orientation– especially when goals are unrealistic or externally imposed– can increase the risk of anxiety, burnout, and psychological distress among athletes (Madigan et al., 2015; Şenel and Ulaş, 2022). For amateur soccer players, balancing goal commitment with emotional intelligence and psychological flexibility is essential for sustaining both well-being and performance (Kotsou et al., 2018; Kaygusuz, 2024). The current literature emphasizes that coaches and sport psychologists should encourage athletes to set realistic, individualized, and achievable goals, supporting them with healthy psychological strategies (Moran, 2016). In this way, goal commitment is associated with personal development and team success the personal development and satisfaction of amateur soccer players but also contributes to the overall success and cohesion of the team.

### 2.3 Mental toughness and sports psychology

Mental toughness (MT) is widely defined as an athlete’s ability to perform at their best under challenging conditions, overcome obstacles, and effectively cope with stress (Jones et al., 2002). In sports psychology, MT is considered a core psychological trait that is associated with success and sustained high performance over time (Mahoney et al., 2014). Although often associated with physical fitness, MT is equally rooted in emotional and cognitive processes, reflecting an athlete’s capacity to remain resilient in the face of both internal and external pressures (Gucciardi et al., 2017, 2021; Kopp et al., 2021). Theoretical frameworks such as the 4Cs model– comprising control, commitment, challenge, and confidence– have been influential in conceptualizing MT (Clough et al., 2002). In this model, control refers to an athlete’s ability to manage emotions and remain composed; commitment involves strong dedication to goals; challenge reflects the perception of obstacles as opportunities for growth; and confidence denotes self-belief and competence. Similarly, Gucciardi et al. (2009) emphasize four main dimensions: self-confidence, control, perseverance, and GC. These dimensions are thought to be associated with athletes’ ability to stay focused, make effective decisions under pressure, and maintain motivation even in the face of adversity such as failure or injury (Mahoney et al., 2014; Kopp et al., 2021).

Recent theoretical perspectives, such as Hardiness Theory (Kobasa, 1979; Maddi, 2006), further enrich our understanding of mental toughness in sports. Hardiness consists of three core components: commitment, control, and challenge. These components help athletes view stressful situations as opportunities for growth rather than threats, maintain a strong sense of purpose, and believe in their ability to influence outcomes. Research shows that athletes with high levels of hardiness are more resilient under pressure, better at coping with adversity, and more likely to sustain motivation and performance over time (Maddi, 2006; Fletcher and Sarkar, 2012; Sarkar and Fletcher, 2014). Integrating hardiness and mental toughness frameworks offers a comprehensive approach to understanding how athletes achieve psychological resilience and success in demanding sporting environments.

Empirical research consistently shows that athletes with high mental toughness (MT) and hardiness are better able to handle competitive stress, recover more quickly from injuries, and sustain long-term performance (Fan and Zhang, 2019; Kopp et al., 2021; Kumbar and Patil, 2024). MT also plays a critical role in maintaining training consistency, focusing under pressure, and adapting to the dynamic demands of team sports. This is especially important for amateur soccer players, who often lack the external motivators, psychological support systems, and resources available to professionals. For these athletes, MT and hardiness are vital for sustaining both individual and team performance (Guszkowska and Wojcik, 2021; Ünlügüzel et al., 2023).

Recent studies highlight that mental toughness (MT) in amateur soccer players is crucial for coping with stress, maintaining motivation, and persisting in training routines (Kumbar and Patil, 2024; Yılmaz and Bostancı, 2024). Additionally, factors such as age, experience, social support, and emotional intelligence (EI) have been identified as important contributors to the development of MT and hardiness (Kotsou et al., 2018). The interaction among MT, hardiness, and EI is gaining attention, as emotionally intelligent athletes are often better at regulating their emotions and adapting to setbacks, which further strengthens their psychological resilience (Laborde et al., 2016, 2018). In summary, MT and hardiness are fundamental determinants of both psychological well-being and sporting success, especially among amateur soccer players. Enhancing these qualities is therefore considered a key strategy for achieving sustainable performance and safeguarding the health and well-being of athletes.

### 2.4 The relationship between emotional intelligence, goal commitment and mental toughness

The relationship between EI, GC, and MT comprises three fundamental psychological components of sports psychology that complement each other and play a crucial role in athletic performance. Recent studies have demonstrated a dynamic and multifaceted interaction among these three concepts (Laborde et al., 2018; Montenegro-Bonilla et al., 2024). Understanding how these psychological structures influence each other, especially in amateur soccer players, is essential for both individual development and team success. GC enables athletes to develop their ability to recognize, understand, and manage their own emotions and those of others. These skills help athletes become more determined and motivated to set and pursue goals (Özcan and Göral, 2020; Orhan and Karagözoğlu, 2021). Athletes with high EI can better analyze the obstacles they encounter in achieving their goals and maintain their motivation by managing emotional fluctuations. Particularly in amateur soccer players, EI has been shown to be associated with higher internal motivation and commitment to goals in situations where external sources of motivation are limited (Acebes Sánchez et al., 2020; Tilindienė, 2024).

Goal commitment (GC) refers to the determination and effort athletes invest in achieving their goals. Athletes with high GC display positive behaviors such as consistent training, persistence in overcoming challenges, and quick recovery from failure (Çekiç and Kurt, 2017; Yılmaz and Bostancı, 2024). Research indicates that GC may mediate the association between EI and MT (Orhan and Ünlü, 2022; Şenel and Ulaş, 2022). In other words, athletes who are highly committed to their goals are more resilient and adaptable when facing stressful and high-pressure situations. This is especially important for amateur footballers in maintaining motivation and mental resilience.

Mental toughness (MT), on the other hand, is the ability of athletes to perform at a high level, overcome obstacles, and cope with stress even under difficult conditions (Jones et al., 2002; Mahoney et al., 2014). Athletes with high MT view the obstacles they encounter in achieving their goals as learning opportunities and can continue on their path without losing motivation in the face of failure (Fan and Zhang, 2019). EI and GC are correlated psychological resources that have been associated with MT. EI helps athletes manage their stress and anxiety levels, while GC provides the perseverance and consistency needed during this process (Laborde et al., 2016, 2018; Montenegro-Bonilla et al., 2024). The interaction among these three concepts is particularly evident in amateur footballers. Compared to professionals, amateur soccer players have more limited resources, support systems, and experiences, so they must develop their psychological resilience largely through their own internal resources. Amateur soccer players with high EI and strong commitment to their goals can improve their psychological resilience and thereby maintain both individual and team performance (Lopes, 2024; Yılmaz and Bostancı, 2024).

### 2.5 The moderating role of demographic variables

The relationships among EI, MT, and GC are influenced by various individual and environmental factors. Demographic variables such as gender, age, sports experience, and educational level can moderate the dynamics among these psychological constructs (Laborde et al., 2016; Kopp et al., 2021; Beniwal and Yadav, 2025). Recent research in sport psychology has increasingly examined how these variables affect both performance and psychological resilience, emphasizing that factors such as social support, cultural background, and competition level also significantly influence these relationships (Kotsou et al., 2018; Guszkowska and Wojcik, 2021). These findings highlight the importance of considering individual differences and contextual factors when developing psychological interventions for athletes.

Emotional intelligence (EI) is defined as a person’s ability to recognize, understand, and regulate their own emotions as well as those of others (Mayer and Salovey, 1997; Mayer et al., 2016). Research consistently shows that EI plays a significant role in athletes’ performance, stress management, and motivation (Lane et al., 2011; Laborde et al., 2016; Campo et al., 2019). Studies have reported that gender can influence EI, with some findings suggesting that female athletes tend to score higher in emotional awareness and empathy than males, although emotion regulation abilities may vary depending on sport type and cultural context (Sánchez-Álvarez et al., 2020; Beniwal and Yadav, 2025). Additionally, recent research indicates that male athletes are often more committed to performance-oriented goals, while female athletes may prefer process-oriented goals. Athletes with higher levels of education tend to have better developed EI skills, and long-term sports experience can enhance emotional control (Roberts et al., 2007; Kopp and Jekauc, 2018, 2025).

Mental toughness (MT) is defined as a person’s ability to remain psychologically resilient in the face of adversity and perform optimally under pressure (Cowden, 2016). Factors such as experience, age, and years of competition have been identified as influential in the development of MT, with more experienced athletes potentially exhibiting higher levels of MT (Gucciardi et al., 2009, 2021; Mahoney et al., 2014). Additionally, social support is important for the development of MT in young athletes, and married athletes may have a more balanced psychological structure (Poucher et al., 2021). Goal commitment (GC) refers to a person’s determination and motivation to achieve their goals (Locke and Latham, 2002, 2013). Some studies have shown that education level and years with sports licenses can lead to differences in individuals’ goal setting and commitment, with athletes who have higher levels of education being more committed to their goals (Weinberg et al., 2001; Amiot et al., 2004). For example, athletes with higher levels of education are more likely to set strategic goals and maintain their motivation despite obstacles (Kristiansen et al., 2012).

In recent years, researchers have increasingly examined the indirect associations of demographic variables with psychological processes using moderation–mediation analyses. In particular, studies in sport psychology have shown that the relationships among EI, GC, and MT can vary depending on individual and environmental factors (Fletcher and Sarkar, 2012; Sarkar and Fletcher, 2014). GC is defined as a person’s ability to recognize, understand, and manage their own emotions and those of others (Mayer and Salovey, 1997), while MT is associated with the ability to remain mentally resilient in the face of challenges and to perform optimally under pressure. GC refers to a person’s determination and motivation to achieve their goals (Locke and Latham, 2002).

The literature indicates that demographic variables can influence the relationships among these three psychological traits in various ways. For example, a study by Rogowska and Tataruch (2024) found that female athletes reported higher levels of GC than their male counterparts, suggesting that GC may serve as a stronger mediating factor in the relationship between EI and MT among women athletes. Similarly, it has been suggested that a long-term sporting career may strengthen the relationship between EI and MT (Swann et al., 2024). Additionally, it has been emphasized that the goal-setting strategies of highly educated athletes may enhance the relationship between EI and toughness (Kristiansen et al., 2012). These findings indicate that models examining moderator and mediator variables together contribute to a better understanding of psychological processes (Hayes, 2022).

**Figure 1:**
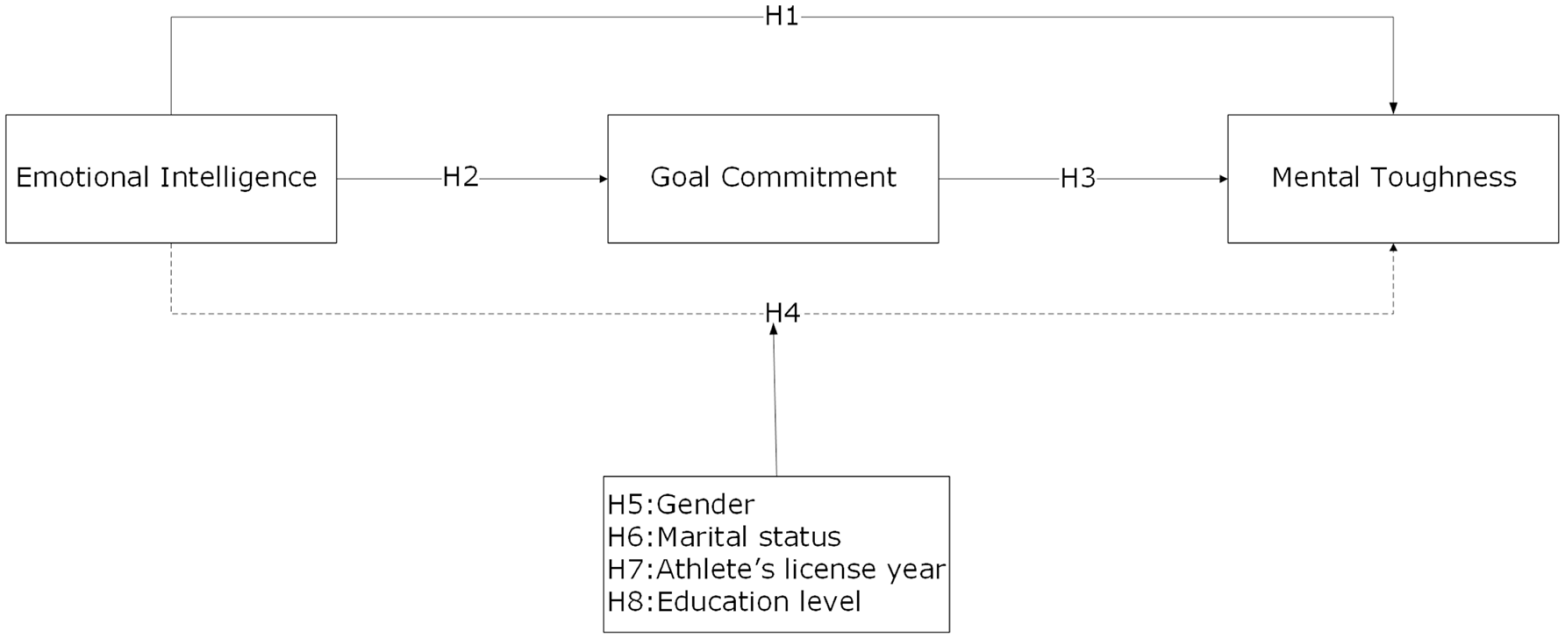
Proposed model of the study.

In this context, the present study investigated the dynamic relationships among EI, GC, and MT in amateur soccer players within a correlational framework. The study examines whether GC mediates the relationship between EI and MT and whether this mediating role is moderated by demographic variables such as gender, marital status, years of sport license, and educational status. Such models provide valuable insights into how psychological structures vary across different demographic groups. They contribute to the literature and support the development of personalized training and psychological support programs in practice (Fletcher and Sarkar, 2012; Sarkar and Fletcher, 2014). Specifically, male and female athletes may demonstrate different psychological processes in the relationship between EI and GC (Levillain et al., 2022; Lepers et al., 2025). More experienced athletes tend to report higher goal commitment (GC), and this pattern co-occurs with a stronger association between EI and MT (Meggs et al., 2016; Gucciardi et al., 2021). Likewise, well-trained athletes’ goal-setting practices are observed alongside a stronger EI–MT association (Lane et al., 2011; Williamson et al., 2022). Therefore, the study will also investigate the regulatory function of GC on the moderating role of these demographic variables in the relationship between EI and MT. Due to the cross-sectional design of the study, the proposed model (Figure 1) and hypotheses were examined within a relational framework. The model and hypotheses of the study are presented below.

Research hypotheses:

*H1*: There is a positive and significant relationship between amateur soccer players’ emotional intelligence and mental toughness.

*H2*: There is a positive and significant relationship between the emotional intelligence of amateur soccer players and their goal commitment.

*H3*: There is a positive and significant relationship between amateur soccer players’ goal commitment and mental toughness.

*H4*: In the relationship between amateur soccer players’ emotional intelligence and mental toughness, goal commitment plays a significant mediating role.

*H5*: Gender significantly moderates the indirect relationship between emotional intelligence and mental toughness via goal commitment.

*H6*: Marital status significantly moderates the indirect relationship between emotional intelligence and mental toughness via goal commitment.

*H7*: Athlete’s license year (experience) significantly moderates the indirect relationship between emotional intelligence and mental toughness via goal commitment.

*H8*: Educational status significantly moderates the indirect relationship between emotional intelligence and mental toughness via goal commitment.

**Figure 2:**
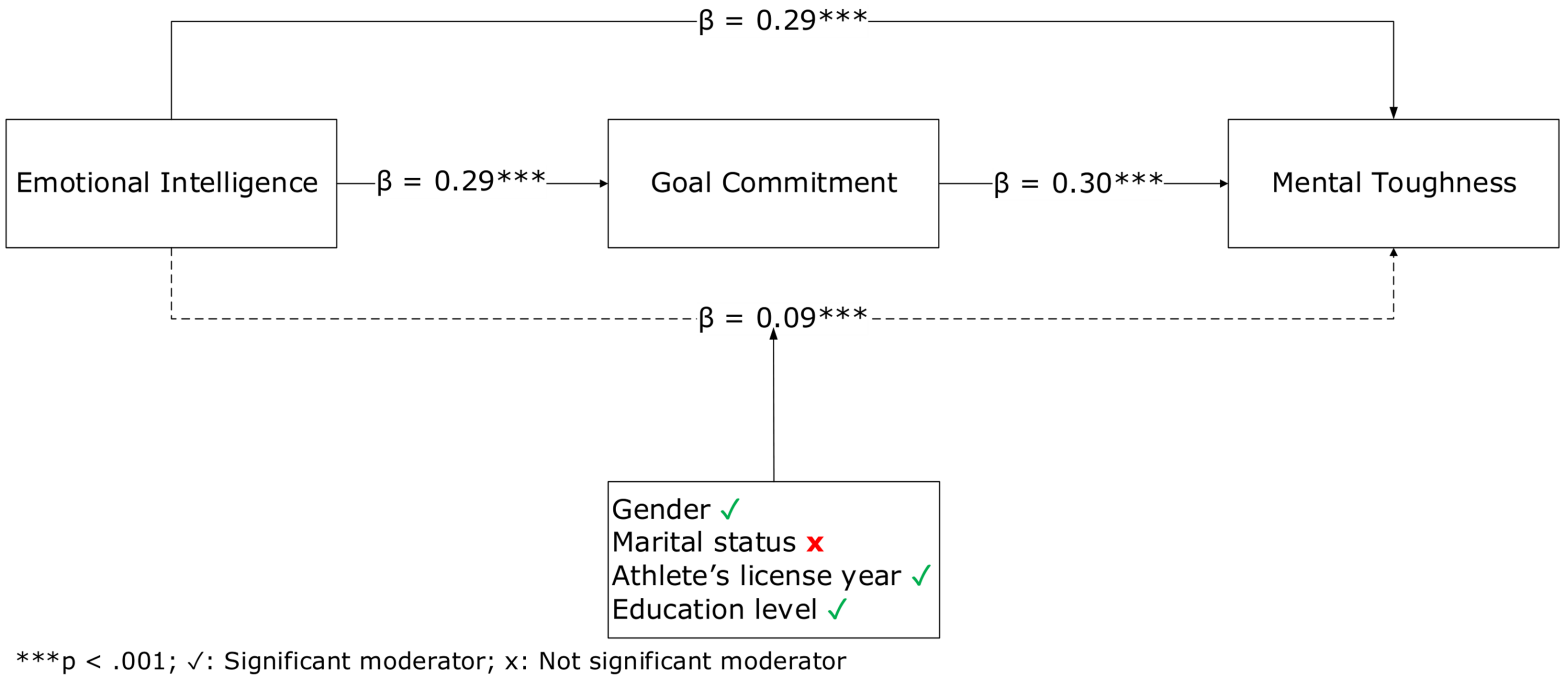
Model of results.

In summary, the strong interaction among EI, GC, and MT provides a comprehensive framework that supports the psychological well-being and athletic performance of athletes. The simultaneous development of these three psychological traits in amateur soccer players is considered an important strategy for sustaining both performance and mental health. In this context, the present study aims to contribute to the literature by examining how the mediating role of GC in the relationship between EI and MT varies across different demographic groups and by providing recommendations for practice (Figure 2).

## 3 Method

### 3.1 Research design

This study employed a cross-sectional correlational design. This approach was selected as an appropriate methodological framework for the primary aim of the research: to examine complex, non-causal relationships—specifically, the mediating role of goal commitment (GC) and the moderating influence of demographic factors (gender, license year, educational status) in the association between emotional intelligence (EI) and mental toughness (MT) at a single time point. We explicitly acknowledge that, although mediation and moderation analyses can help clarify these complex associations, the use of cross-sectional data fundamentally limits the ability to draw causal conclusions or determine temporal ordering (Creswell and Creswell, 2023).

### 3.2 Participants

This study was conducted in Şırnak province, Southeastern Türkiye, during the 2024–2025 soccer season. This region was intentionally selected due to its underrepresentation in sport psychology research, offering a valuable context to examine psychological constructs in a unique socio-cultural environment with limited athletic resources. The sample comprised 262 licensed amateur soccer players, recruited in collaboration with the provincial soccer association. A convenience sampling method was used, inviting all eligible players registered with the association to participate. The inclusion criteria were: (1) holding an active amateur soccer license for at least 1 year, and (2) being an active player in the current season. A total of 456 players met these criteria, and the final sample of 262 represents a participation rate of approximately 57.5%, providing a robust representation of the provincial amateur soccer population. The inclusion of a substantial number of female players (*n* = 74) further strengthened the analytical power for gender-based comparisons.

The participants had a mean age of 18.13 years (SD = 3.89). The sample included 188 males (72%) and 74 females (28%). Regarding demographic characteristics, 223 participants (85%) were single. In terms of licensing experience, 130 (50%) had 1–2 years, 80 (30%) had 3–4 years, and 52 (20%) had 5 or more years. For education, 48 participants (18%) had a primary school diploma, 157 (60%) had a high school diploma, and 57 (22%) had a bachelor’s degree. *A priori* power analysis was conducted using G*Power 3.1.9.7 to justify the sample size (Faul et al., 2009). For a multiple regression analysis with three predictors (modeling the core relationship between EI, GC, and MT), assuming a medium effect size (f^2^ = 0.15), an alpha of 0.05, and a power of 0.95, the minimum required sample size was 77. The obtained sample of 262 participants exceeds this requirement, ensuring sufficient statistical power for the main analyses, including the planned mediation and moderation tests.

### 3.3 Measures

#### 3.3.1 Emotional Intelligence Scale

Emotional intelligence was measured using the 20-item Turkish adaptation of the EI Scale (Kayıhan and Arslan, 2016; original by Lee and Kwak, 2012). The scale uses a 5-point Likert format (1 = Strongly Disagree, 5 = Strongly Agree) and is conceptualized with three dimensions. For this study, the total score was used, ranging from 20 to 100, with higher scores indicating higher EI. The original Turkish adaptation study reported satisfactory psychometric properties, with a Cronbach’s alpha of 0.83 and acceptable fit indices (e.g., CFI = 0.91, RMSEA = 0.075). In the current study, the scale’s validity and reliability for the specific sample were confirmed. A two-step confirmatory factor analysis was conducted to evaluate both the sub-dimensions and the total score. The robust maximum likelihood (MLR) estimator was used due to violations of multivariate normality (Maydeu-Olivares, 2017). The model demonstrated a good fit: χ^2^/df = 2.00, RMSEA = 0.06, CFI = 0.95, TLI = 0.94, SRMR = 0.04. The scale showed high internal consistency, with a Cronbach’s alpha of 0.88 and McDonald’s omega of 0.90. Furthermore, the Average Variance Extracted (AVE) values for the subscales ranged from 0.58 to 0.70, and all Heterotrait-Monotrait Ratio (HTMT) values were below 0.85, confirming convergent and discriminant validity (Kline, 2023).

#### 3.3.2 Goal Commitment Scale

Goal commitment (GC) was assessed using the Turkish adaptation by Şenel and Yıldız (2016) of the scale originally developed by Hollenbeck and Klein (1987) and later reduced to five items by Klein et al. (2014). Responses were recorded on a 5-point Likert scale (1 = Strongly Disagree, 5 = Strongly Agree). Items 1, 2, and 4 were reverse-scored; all items were subsequently recoded so that higher total scores indicate greater GC. The total score ranges from 5 to 25. In the original Turkish adaptation, the scale demonstrated satisfactory psychometric properties, with a Cronbach’s alpha of 0.74 and good model fit (e.g., CFI = 0.97, RMSEA = 0.047). In this study, a confirmatory factor analysis was conducted to validate the scale for the current sample. The model showed excellent fit to the data: χ^2^/df = 1.89, RMSEA = 0.06, CFI = 0.98, TLI = 0.95, SRMR = 0.03. The scale also demonstrated good internal consistency, with a Cronbach’s alpha of 0.81 and McDonald’s omega of 0.81 (Kline, 2023).

#### 3.3.3 Sports Mental Toughness Questionnaire

Mental toughness (MT) was measured using the Turkish adaptation (Altıntaş and Koruç, 2016) of the Sport Mental Toughness Questionnaire, originally developed by Sheard et al. (2009). The inventory consists of 14 items across three sub-dimensions: confidence, perseverance, and control. While the original Turkish adaptation uses a 4-point Likert scale, this study employed a 5-point scale (1 = Completely False, 5 = Completely True) to enhance response sensitivity and variability. The validity of this adapted format was confirmed through the psychometric analyses reported below. In the original Turkish adaptation, the scale demonstrated acceptable psychometric properties, with Cronbach’s alpha coefficients ranging from 0.51 to 0.84 and acceptable model fit (e.g., CFI = 0.91, RMSEA = 0.07). In the current study, the validity and reliability of the 5-point format for this sample were confirmed. A two-step confirmatory factor analysis using the robust maximum likelihood (MLR) estimator showed good model fit: χ^2^(74) = 1.48, RMSEA = 0.04, CFI = 0.97, TLI = 0.97, SRMR = 0.04. The scale showed high internal consistency, with a Cronbach’s alpha of 0.82 and McDonald’s omega of 0.87. The AVE values for the subscales ranged from 0.53 to 0.55, and all HTMT values were below 0.85, confirming convergent and discriminant validity (Kline, 2023). The total score, ranging from 14 to 70, was used for analysis, with higher scores indicating greater MT.

### 3.4 Data collection process

Data were collected between March 10 and May 10, 2024, using an online survey administered through Google Forms. This method was chosen for its practicality, broad reach, and ability to facilitate automatic data recording. To recruit participants, collaboration was established with the provincial soccer association and local coaches, who distributed the survey link to their registered amateur players via team communication channels (e.g., WhatsApp groups). Participation was explicitly voluntary and anonymous; coaches and representatives did not monitor who responded, minimizing potential coercion and ensuring equal access for all players contacted. The survey consisted of two parts: a demographic information section and three main scales (Emotional Intelligence [EI], Goal Commitment [GC], and Mental Toughness [MT]). While the online method ensured efficiency, it may have introduced sampling bias by potentially excluding players with limited internet access or lower digital literacy, possibly skewing the sample toward more tech-comfortable or urban athletes. This limitation is addressed in the discussion section when interpreting the study’s findings.

### 3.5 Ethical issues

This study was conducted in accordance with the ethical principles of the Declaration of Helsinki. Ethical approval was granted by the Batman University Ethics Committee (Decision No: 2024/05–08, Date: 04.07.2024), and official permission was obtained from the Provincial Directorate of Soccer (No: 2024/86, Date: 17.10.2024). All participants were presented with a digital informed consent form at the beginning of the survey. This form detailed the study’s purpose, procedures, and confidentiality measures. Participation required actively checking a box to provide consent; only those who did so could proceed to the survey. A specific procedure was implemented for participants under 18 years of age. After the child provided assent, the survey platform required entry of a parent or guardian’s email address. The system then automatically sent a separate digital consent form to that email. The parentor guardian had to complete this form before the minor could access the main survey, ensuring documented parental permission. To protect confidentiality, the study employed a strict anonymity protocol. No identifying information (such as names, IP addresses, or specific team affiliations) was collected at any point. Data collected through Google Forms was stored on a password-protected server, with access restricted solely to the principal researchers. Technical measures were implemented to prevent multiple submissions from the same device, safeguarding data integrity.

### 3.6 Data analysis

Data analysis was conducted using Jamovi software (version 2.3.28). Confirmatory factor analyses (CFAs) were first performed to verify the construct validity of the scales, with model fit evaluated using standard indices (e.g., χ^2^/df, RMSEA, CFI, SRMR). The reliability and discriminant validity of the scales were confirmed through Cronbach’s *α*, McDonald’s *Ω*, and Average Variance Extracted (AVE) measures. Descriptive statistics and Pearson correlations were calculated to summarize the data and examine bivariate relationships among the main variables. The primary hypotheses were tested using the GLM Mediation Model (medmod) in Jamovi (The Jamovi Project, 2022). Initially, a simple mediation model was tested to determine whether GC mediated the relationship between EI and MT. The moderating roles of demographic variables (gender, license year, educational status) in this mediation model were then examined within the same analytical framework.

Before the analyses, key statistical assumptions were verified, including checks for normality (e.g., Anderson-Darling test), homoscedasticity (e.g., Breusch-Pagan test), and independence of residuals (Durbin-Watson statistic), with no critical violations detected. Multicollinearity was also assessed and ruled out using Variance Inflation Factor (VIF) values. The significance of the indirect and conditional associations was tested using bias-corrected bootstrap confidence intervals based on 5,000 resamples. The model’s explanatory power was evaluated using R^2^, and standardized coefficients (*β*) were interpreted in terms of effect size. Importantly, these analyses identify complex associations and potential pathways but cannot establish causality due to the cross-sectional nature of the data.

## 4 Results

Table 1 presents the descriptive statistics and Pearson correlation coefficients for EI, GC, and MT. Within a 95% confidence interval, the mean scores were as follows: EI (*M* = 77.60, [76.50, 78.70], SD = 9.06), GC (*M* = 20.90, [20.61, 21.20], SD = 2.39), and MT (*M* = 52.69, [51.97, 53.40], SD = 5.89). Skewness and kurtosis values within approximately ±2 are often considered acceptable for normality in applied research (George and Mallery, 2016). Correlation analysis showed positive and significant relationships between EI and GC (r = 0.29, *p* < 0.001) and between EI and MT (*r* = 0.38, *p* < 0.001). Additionally, GC and MT had a moderate positive correlation (*r* = 0.39, *p* < 0.001), confirming the theoretical expectation of positive interrelations among the key study variables.

**Table 1:** Descriptive statistics and correlation between the variables.

Variable	M (95%CI)	SD	Skewness	Kurtosis	1	2
1. Emotional Intelligence (EI)	77.60 (76.50, 78.70)	9.06	−0.34	0.33	–	
2. Goal Commitment (GC)	20.90 (20.61, 21.20)	2.39	−0.98	1.29	0.29^***^	–
3. Mental Toughness (MT)	52.69 (51.97, 53.40)	5.89	−0.35	0.68	0.38^***^	0.39^***^

^***^p < 0.001.

Before conducting mediation analyses, statistical assumptions were examined. The normality assumption was evaluated using the Anderson–Darling test (*A*^2^ = 0.27, *p* > 0.05). Homogeneity of variance was tested using the Goldfeld–Quandt (GQ = 1.00, *p* > 0.05) and Breusch–Pagan (BP = 7.37, *p* > 0.05) tests; variance homogeneity was confirmed by the GQ test, with a minor deviation observed in the BP test. The potential impact of this deviation was minimized by employing bias-corrected bootstrapping with 5,000 resamples. Autocorrelation was checked using the Durbin–Watson test (DW = 1.77, *p* > 0.05), and multicollinearity was assessed through variance inflation factors (VIF = 1.09). These indices indicated that the assumptions for generalized linear modeling were satisfactorily met (Kline, 2023).

The mediation model was tested using a GLM-based approach with bias-corrected bootstrapping (5,000 resamples). Table 2 summarizes the results. GC had a positive and significant total association on MT (*β* = 0.38, *p* < 0.001), explaining 15% of the variance in MT (*R*^2^ = 0.15, *F* (1, 260) = 44.31, *p* < 0.001), supporting H1. GC also significantly associated GC (*β* = 0.29, *p* < 0.001), with EI explaining 8% of the variance in GC (*R*^2^ = 0.08, *F* (1, 260) = 23.76, *p* < 0.001), confirming H2. GC, in turn, had a significant positive relationship on MT (*β* = 0.30, *p* < 0.001), confirming H3. When both EI and GC were entered as variables in the model, they together accounted for 23% of the variance in MT (*R*^2^ = 0.23, *F* (2, 259) = 38.45, *p* < 0.001). The indirect association via GC was significant, (*β* = 0.09, 95% CI [0.05, 0.14], *p* < 0.001), indicating partial mediation (H4 supported). Approximately 24% of the total association (0.09/0.38 × 100 ≈ 24%) was accounted for by the indirect path via GC.

**Table 2:** Mediation analysis results.

Type	Path	B	SE	95% C. I. (a)		β	z	*p*
				LL	UL			
Indirect	EI ⇒ GC ⇒ MT	0.06	0.02	0.03	0.09	0.09	3.56	<0.001
Component	EI ⇒ GC	0.08	0.02	0.05	0.11	0.29	4.84	<0.001
	GC ⇒ MT	0.74	0.17	0.42	1.07	0.30	4.49	<0.001
Direct	EI ⇒ MT	0.19	0.04	0.11	0.27	0.29	4.55	<0.001
Total	EI ⇒ MT	0.25	0.04	0.16	0.34	0.38	5.56	<0.001

EI = Emotional Intelligence; GC = Goal Commitment; MT = Mental Toughness; (a) = 95% confidence intervals were calculated using 5,000 parametric bootstrap samples and refer to unstandardized beta coefficients; betas are fully standardized coefficients.

The moderating roles of demographic variables were further examined within the GLM mediation framework, with conditional indirect associations reported in Table 3. For gender (H5), the indirect association of EI with MT via GC was significant among female athletes (*β* = 0.09, *p* < 0.01) but not among male athletes (*β* = 0.08, *p* > 0.05), indicating moderation by gender. For marital status (H6), the indirect association was significant for both married (*β* = 0.10, *p* < 0.05) and single (*β* = 0.08, *p* < 0.001) participants, suggesting that marital status does not significantly moderate the mediation pathway. For athlete’s license year (H7), the indirect association was significant among athletes with 1–2 years (*β* = 0.07, *p* < 0.05) and ≥ 5 years of experience (*β* = 0.17, *p* < 0.05), but was non-significant for those with 3–4 years of experience (*β* = 0.07, *p* = 0.06), indicating partial support for moderation by sport experience. For educational status (H8), significant indirect associations were found for high school graduates (β = 0.09, p < 0.01) and college graduates (*β* = 0.11, *p* < 0.01), while the path was non-significant for primary school graduates (*β* = 0.06, *p* = 0.19). Collectively, these findings indicate that the indirect association between EI and MT through GC varies by gender, sport experience, and educational level, but not by marital status.

**Table 3:** Moderator analysis results.

Moderator variable	Group	Indirect (EI ⇒ GC ⇒ MT)			β	z	*p*
		Estimate	SE	95%C. I. (a)			
Gender	Male	0.05	0.03	−0.01, 0.11	0.08	1.72	0.08
	Female	0.06	0.02	0.02, 0.10	0.09	3.04	0.00^**^
Marital status	Married	0.07	0.03	0.02, 0.14	0.10	2.22	0.02^*^
	Single	0.05	0.02	0.03, 0.09	0.08	3.33	0.00^***^
Athlete’s license year	1–2 Years	0.05	0.02	0.03, 0.09	0.07	2.06	0.04^*^
	3–4 Years	0.05	0.03	−0.04, 0.10	0.07	1.87	0.06
	5 + Years	0.11	0.05	0.10, 0.21	0.17	2.31	0.02^*^
Education level	Primary School	0.04	0.03	−0.02, 0.09	0.06	1.29	0.19
	High School	0.06	0.02	0.02, 0.10	0.09	2.97	0.00^**^
	Bachelor’s Degree	0.07	0.03	0.07, 0.12	0.11	2.69	0.00^**^

*p < 0.05, **p < 0.01, ***p < 0.001; EI = Emotional Intelligence; GC = Goal Commitment; MT = Mental Toughness; (a) = 95% confidence intervals were calculated using 5,000 parametric bootstrap samples and refer to unstandardized beta coefficients; betas are fully standardized coefficients.

In practical terms, the standardized coefficients (β) were interpreted using conventional benchmarks: β ≈ 0.10 indicates a small effect size, β ≈ 0.30 a medium effect size, and β ≥ 0.50 a large effect size (Cohen, 1988; Funder and Ozer, 2019). The total association between EI and MT (*β* = 0.38) reflects a medium magnitude; the association between EI and GC (*β* = 0.29) is small to medium; and the association between GC and MT (*β* = 0.30) is moderate. The standardized indirect association via GC (*β* = 0.09) represents a small but meaningful practical contribution (Hair et al., 2021). These results suggest that higher EI co-occurs with higher MT, both directly and indirectly via higher GC, and that this pattern varies partially by demographic characteristics.

## 5 Discussion

This study examined the mediating role of GC and the moderating roles of demographic factors (gender, marital status, athlete’s license year, and educational status) in the relationship between EI and MT among amateur soccer players. The findings confirmed a positive and significant association between EI and MT, supporting H1. This result aligns with earlier evidence that athletes with higher EI demonstrate greater resilience, stress tolerance, and motivation regulation (Lin et al., 2017; Zarei et al., 2018; Akgül et al., 2024). These findings are consistent with the view that EI is associated with psychological resources relevant to focus and emotional stability under pressure. Previous studies have emphasized that these traits enhance both adaptive coping and consistency under stress (Laborde et al., 2016; Orhan and Karagözoğlu, 2021; Dosmanova et al., 2024).

The results also showed that EI was positively and significantly associated with GC (supporting H2). Athletes with higher EI seem more capable of maintaining motivation, demonstrating perseverance, and regulating emotional responses when facing adversity. These findings align with previous research indicating that emotionally intelligent individuals exhibit higher levels of self-discipline and sustained focus on personal goals (Peña-Sarrionandia et al., 2015; Usán Supervía and Quílez Robres, 2021). GC has been linked not only to effective goal pursuit but also to flexible goal adjustment in changing environments (Mayer et al., 2004; Goleman, 2005). These results are consistent with earlier findings suggesting that emotion regulation, self-awareness, and empathy– key components of EI– facilitate persistence and constructive responses to setbacks in sport (Goleman, 1998; Kopp and Jekauc, 2018).

Furthermore, the positive link between GC and MT (H3 supported) shows that athletes who remain committed to their goals tend to display greater psychological endurance and self-regulation. This pattern aligns with the conceptual frameworks of hardiness (Kobasa, 1979) and toughness (Maddi, 2006), both of which emphasize perseverance as a key psychological determinant of mental resilience. Empirical research reinforces this connection: athletes with higher GC perform better under competitive stress and exhibit stronger coping resources (Coulter et al., 2018; Gucciardi et al., 2021). Meta-analytic findings also confirm robust associations between MT and goal striving across team sports (Pandian et al., 2023). In addition, recent research suggests that mindfulness-based interventions can further enhance mental toughness by promoting goal-directed mindfulness, particularly in amateur soccer players (Wang, 2023). Together, these findings underscore that maintaining clear goals strengthens psychological durability and contributes to performance consistency in amateur soccer.

The mediation analysis results support H4, indicating that GC partially mediates the association between EI and MT. Approximately one-quarter of the total association between EI and MT is accounted for via GC. This pattern is consistent with emotionally intelligent athletes showing higher resilience-related attributes, with goal commitment representing a potential correlational pathway. This finding aligns with the theoretical propositions of Mayer and Salovey’s (1997) EI model and Fredrickson’s (2001) broaden-and-build theory, which emphasize that positive emotion and emotional clarity promote motivation and cognitive flexibility. Empirical evidence also supports this pathway– emotionally intelligent athletes demonstrate stronger motivational focus, self-regulatory skills, and social adaptiveness, which enhance both their GC and toughness (Cowden, 2016; Laborde et al., 2018; Mitić et al., 2020; Montenegro-Bonilla et al., 2024).

Importantly, the present results also show that this mediating relationship is moderated by certain demographic factors, supporting H5–H8. Gender, athlete experience (license year), and educational status significantly influenced the indirect relationship between EI and MT via GC, while marital status did not. Specifically, the mediating role appeared stronger among female athletes, suggesting that the EI–GC–MT associations may be more pronounced among women. These results corroborate research suggesting that female athletes often score higher in EI and display stronger associations between emotional awareness and sport performance (Kopp and Jekauc, 2018; Rubio et al., 2022; Ayılgan et al., 2023). Such gender differences may reflect heightened emotional sensitivity and empathy, enabling female athletes to translate emotional awareness into motivational persistence (Campos-Uscanga et al., 2023).

Athlete experience also moderated the indirect association. The EI–GC–MT linkage was strongest among athletes with longer sport experience, aligning with work suggesting that emotional and motivational competencies tend to accumulate with competitive exposure (Castro-Sánchez et al., 2018; Montenegro-Bonilla et al., 2024). With increased experience, athletes often report more consistent goal-setting habits and emotional coping strategies, which co-occur with stronger resilience (Laborde et al., 2016; Quinn and Cavanaugh, 2017). This pattern suggests that athletic experience may provide a psychological context in which EI is more strongly associated with MT.

Educational level further influenced the mediation pathway, as athletes with higher education levels exhibited stronger indirect relationships between EI and MT. This finding supports previous research linking cognitive flexibility and metacognitive skills to both self-regulation and psychological resilience (Levy et al., 2012; Rodrigues et al., 2024). Educational environments often promote reflection, problem solving, and social interaction, all of which contribute to goal clarity and persistence (McKay et al., 2023; Yarayan et al., 2018). In contrast, lower educational levels may restrict access to these cognitive-emotional resources, making it more difficult for athletes to maintain commitment and MT over time (Şenel and Ulaş, 2022).

The non-significant moderation by marital status indicates that relationship status appears to have a limited association with emotional–motivational processes in sport. Consistent with prior research, structural and relational factors such as the coach-athlete relationship, team cohesion, and support networks appear to have a stronger impact than marital context on athletes’ psychological outcomes (Davis et al., 2013; Stewart et al., 2024). Overall, the moderation results extend previous evidence by clarifying that demographic variables shape how EI translates into resilience– through an intermediate pathway of GC– among amateur soccer players in Türkiye. Together, these findings advance understanding of how EI indirectly enhances MT and demonstrate that this process is both motivationally and contextually dependent. They provide empirical support for contemporary models of psychological resilience in sport, which view emotional and motivational mechanisms as interactive rather than independent processes (Gucciardi et al., 2021; Pandian et al., 2023; Shi et al., 2025).

## 6 Limitations and recommendations for future research

These findings should be interpreted in light of several limitations. First, data were collected exclusively from amateur soccer players in a single province of Türkiye, which limits the external validity and generalizability of the results. Cultural, socioeconomic, and environmental differences may influence emotional intelligence (EI) and psychological adaptation in athletes. Second, the cross-sectional design precludes causal inferences; therefore, experimental or longitudinal studies are needed to examine how goal commitment (GC) and EI co-evolve over time and how their associations with mental toughness (MT) develop. Third, reliance on self-report measures introduces the possibility of social desirability and common-method bias. Future research should use multi-method approaches, such as coach ratings, peer evaluations, and objective performance indicators. Additionally, online data collection may have limited participation from athletes with low digital accessibility, potentially introducing participation bias. Expanding research to include multiple sports, competitive levels, and regions would enhance representativeness and allow for comparative analyses between amateur and professional populations. In future studies, a multi-region, multi-level, and cross-cultural sampling strategy should be adopted to enhance external validity and representativeness.

Future studies should also consider additional contextual factors, such as team climate, perceived coaching style, and motivational environment, which may further influence the emotional and goal-directed processes contributing to MT. Longitudinal and intervention-based designs could more robustly assess causal mechanisms and test specific training programs aimed at developing EI, self-regulation, and goal management skills.

## 7 Conclusion

### 7.1 Theoretical conclusions

The present study provides empirical evidence that the relationship between EI and MT among amateur soccer players is partially mediated by GC and differentially moderated by demographic factors– specifically, gender, athlete’s license year, and educational status. These findings enrich theoretical models of EI by demonstrating that GC may function as a motivational bridge in the observed associations between emotional competence and resilience, reflecting both psychological and contextual dynamics in athletic performance. The results also highlight the importance of individual differences in shaping how EI functions within the broader framework of MT and motivation in sport.

### 7.2 Practical conclusions

For practitioners, these findings highlight the importance of consider integrating EI-focused and structured goal-setting practices. Short, targeted interventions that combine emotion regulation, self-awareness, and goal management training may strengthen both goal commitment and mental toughness in amateur soccer players. Tailoring these programs to gender, experience, and educational background may be associated with better engagement and perceived usefulness. Implementing mixed-method monitoring systems, such as combining athlete self-assessments, coach evaluations, and simple performance metrics, can help evaluate progress and optimize developmental outcomes in real-world club environments.

## Data Availability

The raw data supporting the conclusions of this article will be made available by the authors, without undue reservation.
